# Quality assessment tests for tumorigenicity of human iPS cell-derived cartilage

**DOI:** 10.1038/s41598-020-69641-4

**Published:** 2020-07-30

**Authors:** Yoshiaki Takei, Miho Morioka, Akihiro Yamashita, Tomohito Kobayashi, Nobuyuki Shima, Noriyuki Tsumaki

**Affiliations:** 1 Cell Induction and Regulation Field, Department of Clinical Application, Center for iPS Cell Research and Application, Kyoto University, 53 Kawahara-cho, Shogoin, Sakyo-ku, Kyoto, 606-8507 Japan; 2 Regenerative Medicine Technology Department, Healthcare R&D Center, Asahi KASEI corporation, Tokyo, Japan

**Keywords:** Osteoarthritis, Translational research, Stem-cell research, Induced pluripotent stem cells

## Abstract

Articular cartilage damage does not heal spontaneously and causes joint dysfunction. The implantation of induced pluripotent stem cell (iPSC)-derived cartilage (iPS-Cart) is one candidate treatment to regenerate the damaged cartilage. However, concerns of tumorigenicity are associated with iPS-Cart, because the iPSC reprogramming process and long culture time for cartilage induction could increase the chance of malignancy. We evaluated the tumorigenic risks of iPS-Cart using HeLa cells as the reference. Spike tests revealed that contamination with 100 HeLa cells in 150 mg of iPS-Cart accelerated the cell growth rate. On the other hand, 150 mg of iPS-Cart without HeLa cells reached growth arrest and senescence after culture, suggesting less than 100 tumorigenic cells, assuming they behave like HeLa cells, contaminated iPS-Cart. The implantation of 10,000 or fewer HeLa cells into joint surface defects in the knee joint of nude rat did not cause tumor formation. These in vitro and in vivo studies collectively suggest that the implantation of 15 g or less iPS-Cart in the knee joint does not risk tumor formation if assuming that the tumorigenic cells in iPS-Cart are equivalent to HeLa cells and that nude rat knee joints are comparable to human knee joints in terms of tumorigenicity. However, considering the limited immunodeficiency of nude rats, the clinical amount of iPS-Cart for implantation needs to be determined cautiously.

## Introduction

Cartilage consists of chondrocytes embedded in abundant cartilage extracellular matrix (ECM). Articular cartilage covers the ends of bones and constitutes the joint surface, providing lubrication in diarthrodial joints. Articular cartilage has only limited capacity for intrinsic repair, and its damage due to trauma hardly heals, leading to debilitating conditions such as osteoarthritis. The implantation of cells that can induce cartilage is an expected treatment against the focal damage of articular cartilage. Cell sources for such cell therapy include chondrocytes, mesenchymal stromal/stem cells (MSCs), and pluripotent stem cells (PSCs). Autologous chondrocytes are limited in number, and thus need to be expanded in culture prior to implantation, which causes the loss of their chondrocytic properties^1^. On the other hand, MSCs and PSCs are subjected to expansion and chondrogenic differentiation before implantation.

Induced PSCs (iPSCs) are somatic cells that have been reprogrammed to a PSC state, but unlike other PSCs such as embryonic stem cells (ESCs), they are free from ethical issues such as the sacrifice of embryos. Clinically applicable iPSCs have been produced by the transient expression of reprogramming factors using episomal vectors, and the resultant iPSCs are free from footprints.

Laboratories including ours reported protocols for the creation of cartilage from human iPSCs^2^. In these protocols, iPSCs were differentiated to chondrocytes, followed by transfer to 3D culture to make the chondrocytes produce ECM for the creation of iPSC-derived cartilage (iPS-Cart). Cartilage and iPS-Cart have low immunogenicity^3–5^, and cartilage has thus been transplanted in an allogeneic manner^4,6–9^. Although the implantation of allogeneic iPS-Cart into the damaged region is expected to treat patients with focal damage in articular cartilage, the risk of tumor formation by the implants must be assessed before moving to clinical application.

Cultured chondrocytes and MSCs has been clinically implanted into joints to treat articular cartilage damage without tumor formation after the implantation. On the other hand, iPSCs have two specific aspects that raise the risk of tumor formation. One is residual undifferentiated iPSCs in iPS-Cart even after chondrogenic differentiation, which can produce teratoma after implantation. The other is the long culture period needed to induce cartilage from PSCs and the process of cell reprogramming during the generation of iPSCs, which are associated with an increased probability of iPS-Cart cells becoming malignant (transformed cells). Therefore, quality assessment systems to monitor tumorigenic cellular impurities should be established^10^ and applied to iPS-Cart in order to judge whether iPS-Cart is safe for clinical use. Tumorigenicity assays have been reported for cardiomyocytes^11^ and retinal cells^12^ derived from iPSCs, but not for chondrocytes or cartilage derived from iPSCs.

The number of undifferentiated iPSCs and transformed cells contaminating iPS-Cart can be estimated by separate tests. The contamination of undifferentiated iPSCs needs to be estimated by analyzing the expression of iPSC-specific genes including LIN28A (manuscript in preparation) and spike tests in which iPS-Cart are mixed with undifferentiated iPSCs and subjected to analyses. In the current study, we focused on transformed cells and established a system to estimate their number in iPS-Cart. We tried to quantify the number of transformed cells in iPS-Cart by a spike test in vitro and to estimate the maximum number of transformed cells that does not produce tumors in knee joints by transplantation into the nude rat knee joints in vivo. For this purpose, we need to select the appropriate cell type to be used for spike tests and transplantation experiments. Because all types of malignant cell could theoretically develop from iPSCs due to pluripotency, it is difficult to select one cell type. Among the various malignant cell lines, we chose HeLa cells, because they are one of most widely used and analyzed among tumorigenic cell lines and because as a few as 10 HeLa cells can form tumors in subcutaneous tissue^13^. Assuming that the tumorigenic cells in iPS-Cart are equivalent to HeLa cells and that nude rat knee joints are comparable to human knee joints in terms of tumorigenicity, we built a rationale to estimate the maximum amount of iPS-Cart that would not produce tumors caused by transformed cells after the implantation. The rationale of this system will be helpful for assessing the tumorigenic properties caused by transformed cells in other cell-processed therapeutic products that are to be implanted into knee joints.

## Results and discussion

### Preparation of QHJI iPS-Cart

**Figure 1:**
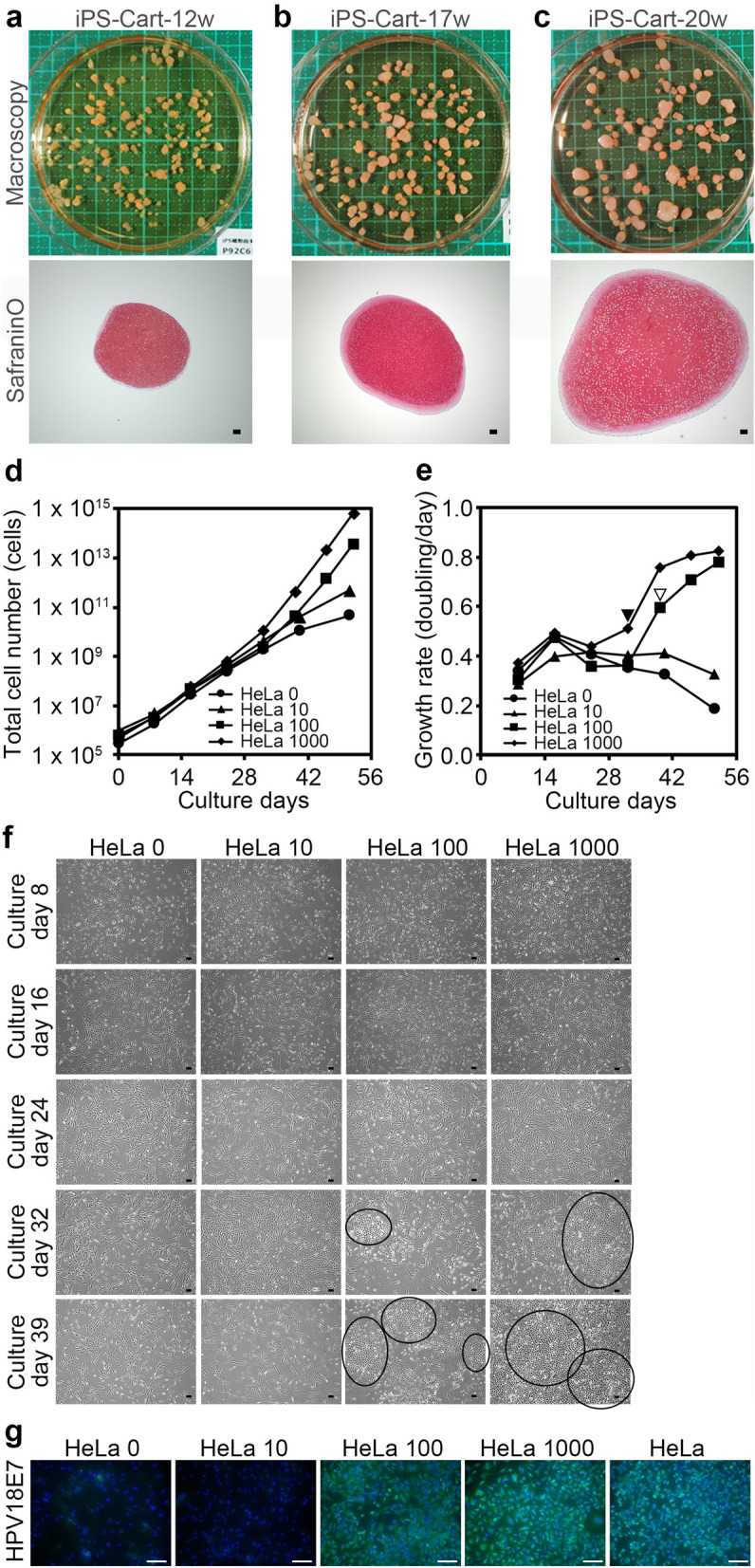
Cell growth analysis for the detection of tumorigenic cells in 30 mg QHJI iPS-Cart. 30 mg iPS-Cart-12w were mixed with none, 10, 100, or 1,000 HeLa cells (respectively designated as HeLa 0, HeLa 10, HeLa 100, and HeLa 1,000) and digested with collagenase to release the cells, which were plated on dishes, cultured, and passaged. Cell numbers were counted with every passage. (**a**–**c**) Appearance and histology of QHJI iPS-Cart-12w, -17w, and -20w. Safranin O-fast green-iron hematoxylin staining. Scale bars, 100 μm. (**d**) Total number of iPS-Cart cells spiked with or without HeLa cells. (**e**) Growth rate of iPS-Cart cells spiked with or without HeLa cells. (**f**) Images of the cell culture on different days after the start of the culture. Circles denote colonies of small angular cells. Scale bars, 100 μm. (**g**) Immunocytochemistry using anti-HPV18E7 antibody (green) at day 40. Scale bars, 100 μm. Blue color is DAPI.

We created iPS-Cart by chondrogenically differentiating QHJI, a clinical grade iPSC line, for 12 weeks. iPS-Cart was used for experiments immediately (designated as iPS-Cart-12w) or after maintaining them in chondrogenic medium for 5 or 8 weeks (designated as iPS-Cart-17w or iPS-Cart-20w, respectively). iPS-Cart-12w, -17w, and -20w were particles 2–3 mm in size. Histological analysis indicated that iPS-Cart were cartilaginous, as the cells were embedded in ECM that was positively stained with safranin O (Fig. 1a–c), consistent with previous reports^14,15^.

### Cell growth analysis for detection of tumorigenic cells in iPS-Cart-12w

Because tumorigenic cells show faster growth than somatic cells and do not show an attenuation of growth after serial passages^16–18^, cell growth analysis can be used to detect the presence of tumorigenic cells in culture^19,20^. For the cell growth analysis on QHJI iPS-Cart, we added 10, 100, or 1,000 HeLa cells to 30 mg of QHJI iPS-Cart-12w (respectively designated as HeLa 10, HeLa 100, and HeLa 1,000) and compared their growth with that of iPS-Cart-12w alone (HeLa 0). Since iPS-Cart is tissue consisting of cells embedded in cartilage ECM, we subjected iPS-Cart to digestion with collagenase to release the iPS-Cart cells, which were then plated on dishes.

Cell growth analysis indicated that cell numbers increased constantly for up to 32 days and 40 days and more slowly after those times in culture with HeLa 0 and HeLa 10, respectively (Fig. 1d). On the other hand, the increase was accelerated with HeLa 100 and HeLa 1,000 after around 32–39 days of culture (Fig. 1d). Cell growth rate (doubling/day) analysis revealed that the growth rates of HeLa 100 and HeLa 1,000 were higher after 39 days (Fig. 1e, open arrowhead) and 32 days (Fig. 1e, closed arrowhead) of culture, respectively, whereas those of HeLa 0 and HeLa 10 began to decrease around these times (Fig. 1e). These results suggest that the population of HeLa cells increased in a dose- and time-dependent manner. The growth rate of HeLa 1,000 reached a plateau at 46 days after the start of culture, which probably corresponds to the growth rate of HeLa cells.

Close observation of the culture revealed a colony formation of small cells with an angular shape in HeLa 100 and HeLa 1,000 at 32 and 39 days of culture, respectively (Fig. 1f, circles). The sizes of the colonies increased with the culture period. Cells in these colonies specifically expressed HPV18E7, a specific marker for HeLa cells (Fig. 1g). These results suggest that the inoculated HeLa cells were responsible for the accelerated increase of cell numbers in HeLa 100 and HeLa 1,000.

The data collectively suggest that the cell growth analysis can detect the presence of 100 or more tumorigenic cells in 30 mg iPS-Cart-12w if assuming these tumorigenic cells have growth properties comparable to those of HeLa cells.

### Improving sensitivity of cell growth analysis for detection of tumorigenic cells in iPS-Cart-12w

**Figure 2:**
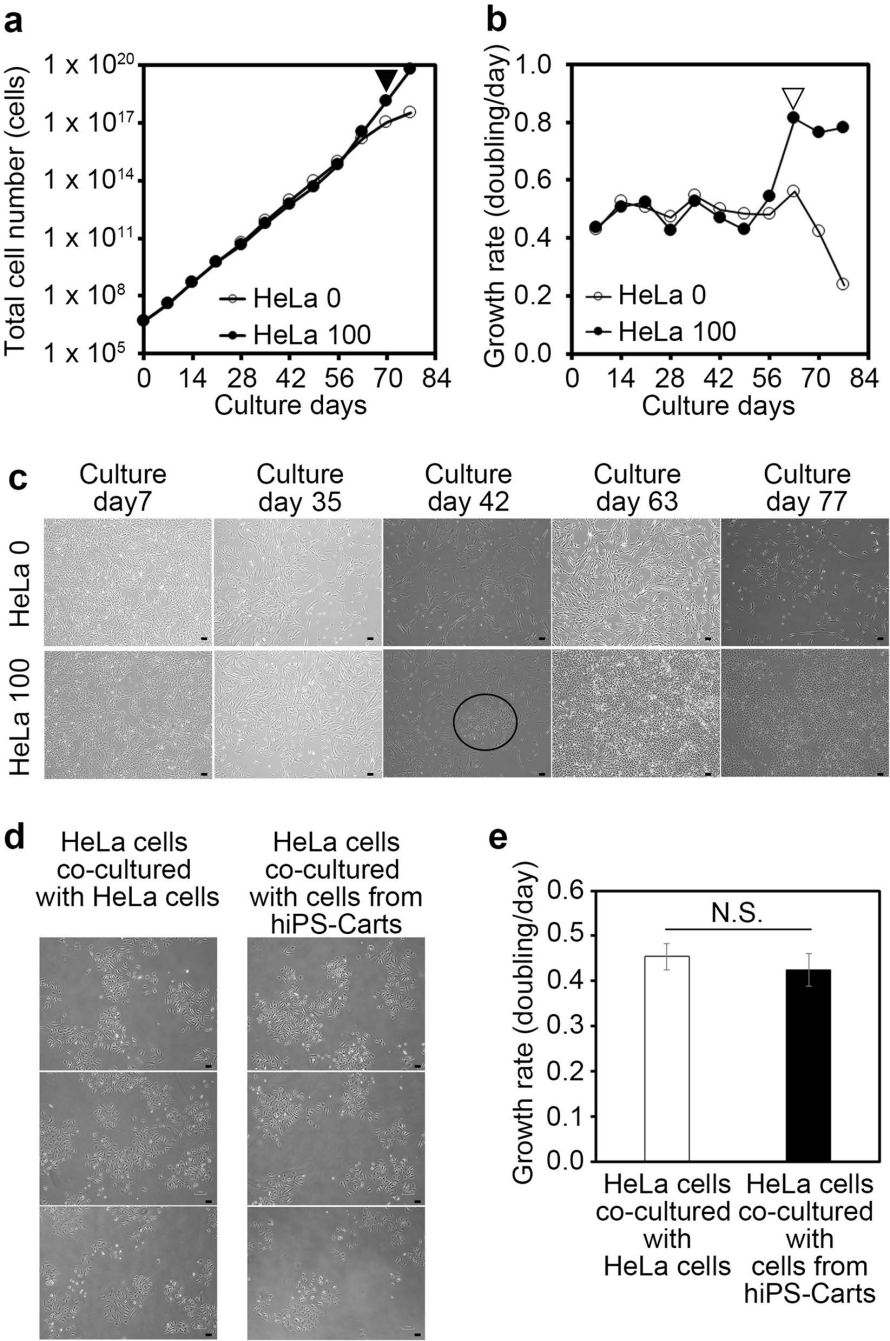
Cell growth analysis for the detection of tumorigenic cells in 150 mg QHJI iPS-Cart. 150 mg of iPS-Cart were mixed with 100 HeLa cells and digested with collagenase to release the cells, which were plated on dishes, cultured, and passaged. Cell numbers were counted with each passage. (**a**) Total number of iPS-Cart cells spiked with HeLa cells. Three dishes were cultured and counted six times for each sample. (**b**) Growth rate of iPS-Cart cells spiked with HeLa cells. (**c**) Images of the cells on different culture days. The circle denotes a colony of small angular cells. Scale bars, 100 μm. (**d**, **e**) HeLa cells were co-cultured with HeLa cells or cells isolated from hiPS-Cart. (**d**) Microscopic images of HeLa cells. Scale bars, 100 μm. (**e**) The number of cells was counted after 5 days of culture. n = 3 dishes. P > 0.05 by Student’s t-test.

We next examined whether the sensitivity of the test can be improved by increasing the amount of iPS-Cart used in the experiment. We added none or 100 HeLa cells to 150 mg of iPS-Cart-12w, digested the iPS-Cart with collagenase, and performed cell growth analysis (Fig. 2a) and cell growth rate analysis (Fig. 2b). A colony formation of small cells with an angular shape was found at 42 days (Fig. 2c, circle), and the cell growth rate increased at 49 days after the start of culture and reached a plateau at 63 days (Fig. 2a, closed arrowhead; Fig. 2b, open arrow head) in HeLa 100. For HeLa 0, the cell growth cell rate behaved constantly until 63 days and decreased thereafter. The results suggest that the test can detect the presence of 100 tumorigenic cells in 150 mg iPS-Cart-12w if again assuming these tumorigenic cells have growth properties comparable to those of HeLa cells. The results and assumption also indicate that if 150 mg QHJI iPS-Cart-12w contains HeLa cell-equivalent tumorigenic cells, their number is less than 100.

To examine whether the interaction of HeLa cells and iPS-Cart cells affects the proliferation rate of HeLa cells, we co-cultured HeLa cells and iPS-Cart cells but found no significant effect on the rate (Fig. 2d,e).

### Confirmation of cell senescence in iPS-Cart

**Figure 3:**
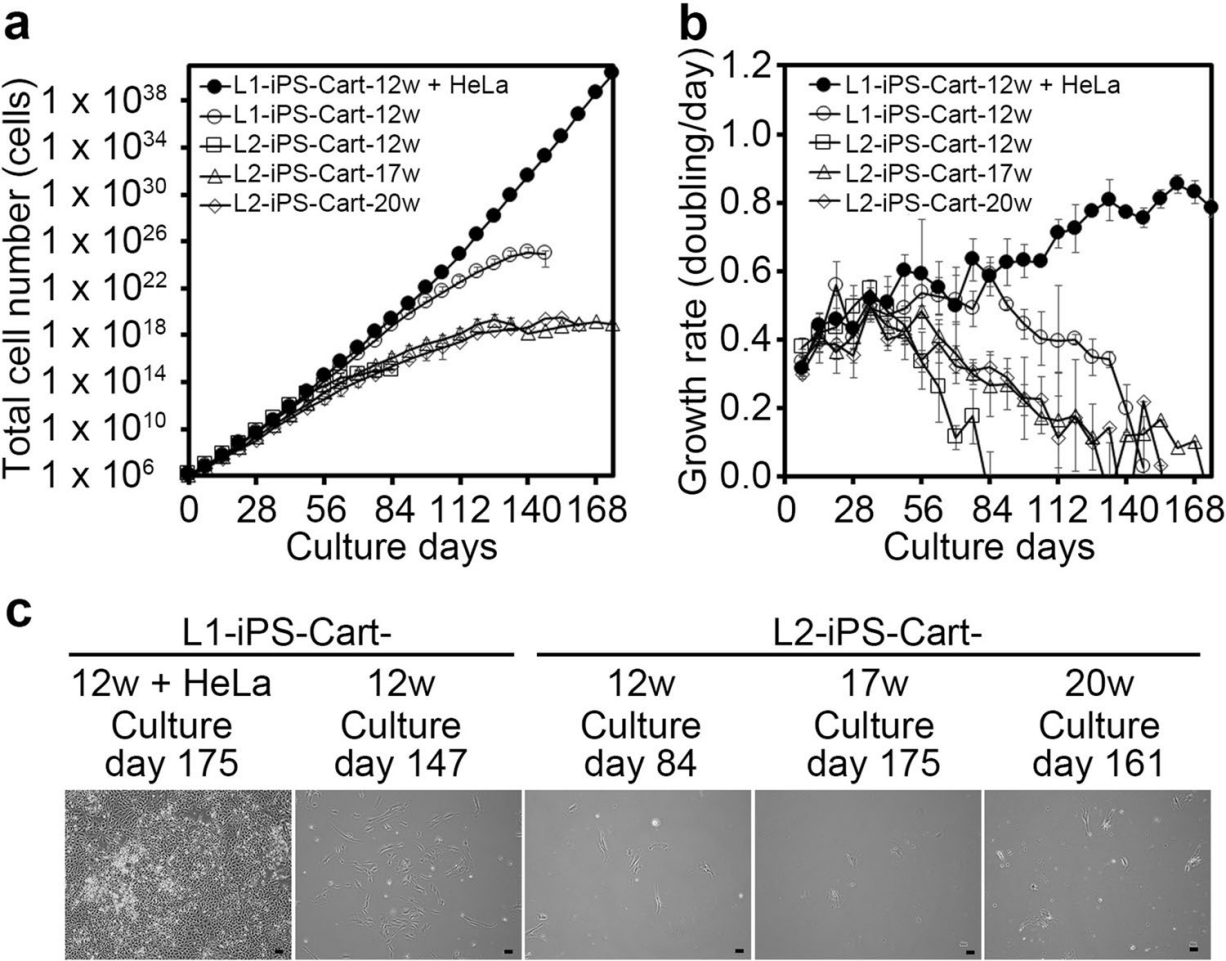
Cell growth analysis of iPS-Cart cells. 30 mg each of L1-iPS-Cart-12w, L2-iPS-Cart-12w, L2-iPS-Cart-17w, and L2-iPS-Cart-20w were digested with collagenase to release the cells, which were plated on dishes, cultured, and passaged for expansion. Cell numbers were counted at passages. As a positive control for immortality, L1-iPS-Cart-12w was mixed with 100 HeLa cells and processed similarly. (**a**) Total number of iPS-Cart cells. (**b**) Growth rate of iPS-Cart cells. (**c**) Images of the cells on the culture days on which cell growth stopped. Scale bars, 100 μm.

Cellular immortalization is one of the prerequisite steps in tumorigenesis. The European Medicines Agency stated that the evaluation of in vitro cell senescence after serial passaging is sufficient to prove the absence of immortalized/tumorigenic cells in human somatic cell-based products^21^. Therefore, we examined the cell senescence in iPS-Cart. We subjected cells released from 30 mg iPS-Cart by digestion with collagenase to longer culture time and more passages. We tested two lots of iPS-Cart (L1-iPS-Cart and L2-iPS-Cart), which were respectively created from two different stock tubes of QHJI iPSCs, to examine reproducibility. In addition, to assess whether the maintenance culture of iPS-Cart in chondrogenic medium contributes to cellular immortalization, we tested iPS-Cart-17w and -20w. As a positive control for immortalization, L1-iPS-Cart-12w spiked with 100 HeLa cells showed an accelerated increase in the total cell number (Fig. 3a) and increased growth rate beyond 84 days of culture (Fig. 3b). On the other hand, a cell growth analysis of all samples of iPS-Cart in the absence of HeLa cells (L1-iPS-Cart-12w, L2-iPS-Cart-12w, L2-iPS-Cart-17w, and L2-iPS-Cart-20w) showed that the total cell numbers initially increased steadily, but then slowed and finally reached a plateau (Fig. 3a). The cell growth rate (doubling/day) analysis of these samples revealed growth rates that gradually decreased with time and finally reached zero within 84 to 175 days from the start of the culture (Fig. 3b). The periods necessary for reaching senescence varied more between experimental lots than between the different periods of maintenance culture. At this time, the cells showed flat morphology, which is typical of senescent cells (Fig. 3c).

**Figure 4:**
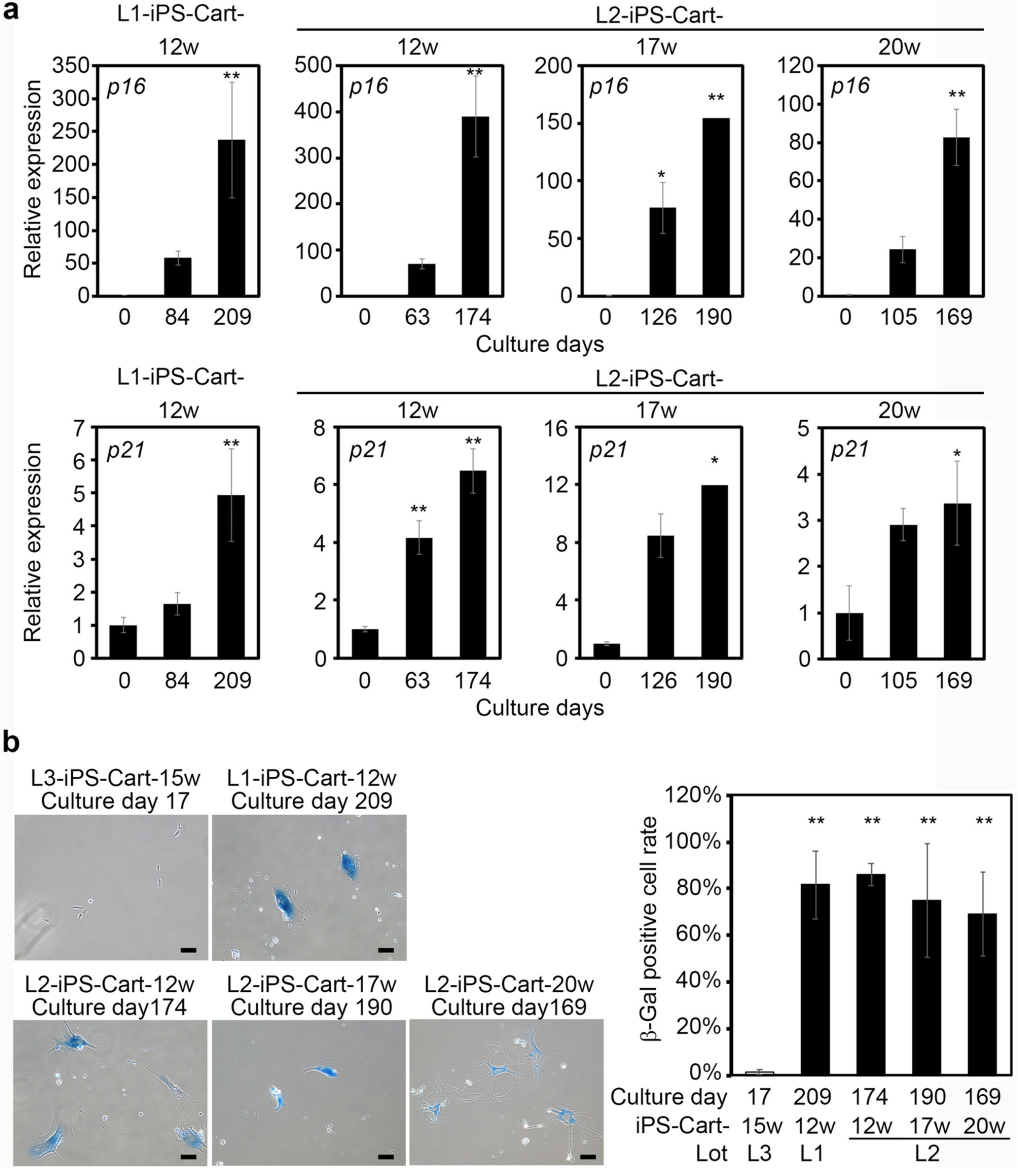
Expression analysis of senescence markers after prolonged culture of cells from iPS-Cart. (**a**) Real-time RT-PCR expression analysis for *p16* (top row) and *p21* (bottom row). Cells from iPS-Cart were expanded in culture for the period indicated, and RNA was extracted. *P < 0.05 and **P < 0.01 versus 0 days by one-way ANOVA with Tukey`s HSD test. n = 3 samples except for the cells collected after 190 days culture of cells from L2-iPS-Cart-17, in which n = 2. (**b**) Cells from iPS-Cart were expanded for the periods indicated and subjected to β-galactosidase staining. Cells from L3-iPS-Cart-15w were cultured for 15 days and served as a negative control. *Left*, Images of β-galactosidase staining. *Right*, The number of β-galactosidase-positive cells per total cells were calculated. **P < 0.01 compared to the negative control by one-way ANOVA with Tukey`s HSD test. n = 3 samples.

To further confirm that the cells had reached senescence, we examined senescence markers. Cells from L1-iPS-Cart-12w, L2-iPS-Cart-12w, L2-iPS-Cart-17w, and L2-iPS-Cart-20w showed increased expression levels of p16 and p21 with increased culture time (Fig. 4a). Prolonged culture also caused the expression of senescence-associated β-gal activity (Fig. 4b), an indicator of cellular senescence. These results collectively suggest that cells from hiPS-Cart do not contain immortalized/tumorigenic cells and reach senescence.

### Number of cells needed for HeLa cells to form tumors in knee joints

Next, we investigated the minimum number of tumorigenic cells necessary to form tumors in knee joints, the site into which we are planning to implant iPS-Cart in future clinical tests. This number depends on the behavior of the tumorigenic cells and the local environment of the implantation site. However, we cannot know the behavior of the tumorigenic cells, because any type of cell can theoretically emerge from iPSC-derived cartilage due to the pluripotency of iPSCs. Therefore, we used HeLa cells for these experiments, because HeLa cells are well known and have been widely tested for their tumorigenicity^22^. The World Health Organization (WHO) recommends HeLa cells to be used as the positive control in common tumorigenicity tests with cell substrates for biological products^22^.

**Figure 5:**
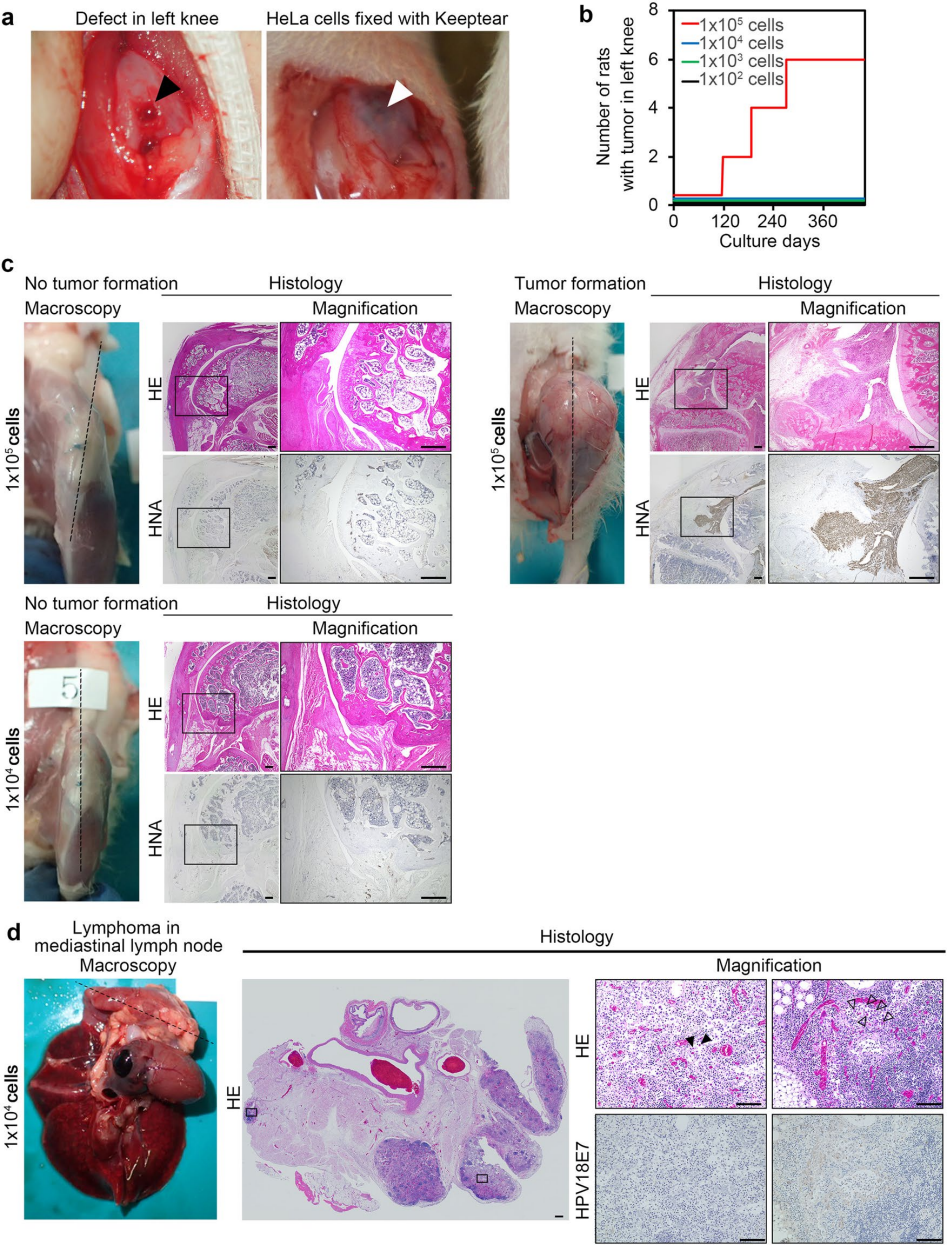
Tumor formation after implantation of various numbers of HeLa cells into joint surface defects in the left knee joints of nude rats. (**a**) Images of joint surface defects before (left, black arrowhead) and after (right, white arrowhead) the implantation of HeLa cells with atelocollagen gel (keeptear). (**b**) Tumor incidence of HeLa cells. The relationship between the dose and the tumorigenic incidence of HeLa cells is shown. N = 8 nude rats in each group (4 male and 4 female). (**c**) Gross appearance after the removal of skin and histology of left knee joints from three representative nude rats. Histological semi-serial sections were stained with HE and immunostained with anti-human nuclear antigen (HNA) antibody. *Top left*, the rat knee that received 1 × 10^5^ HeLa cells did not develop tumors. *Top right*, the rat knee that received 1 × 10^5^ HeLa cells developed tumors. *Bottom*, the rat knee that received 1 × 10^4^ HeLa cells did not develop tumors. (**d**) Gross appearance and histology of lymphoma in the mediastinal lymph node formed in one nude rat that received 1 × 10^4^ HeLa cells 427 days after the implantation. Histological semi-serial sections were stained with HE and immunostained with anti-HPV18E7 antibody. Scale bars, 500 μm.

Since we plan to implant iPS-Cart into the defects of the knee joint surface in future clinical tests, we created defects in the knee joint surface of nude rats, which are the largest available immunodeficient animals. We implanted various numbers of HeLa cells into the defects (Fig. 5a) and observed the rats for 460 days, which approximates their life span. No rats out of four male and four female rats that received up to 1 × 10^4^ HeLa cells developed tumors in the left knee joints (Fig. 5b, Table 1). On the other hand, 3 out of 4 male and 3 out of 4 female rats that received 1 × 10^5^ HeLa cells developed tumors in the left knee joints (Fig. 5b,c). One nude rat that received 1 × 10^4^ HeLa cells developed lymphoma in the mediastinal lymph node, but immunohistochemical analysis did not detect the expression of HPV18E7, which is specifically expressed in HeLa cells (Fig. 5d, Table 1), indicating that HeLa cells were not the cause of the tumor formation. Tumors are known to develop spontaneously in rats^23–25^, which could explain the lymphoma. These results collectively suggest that malignant cells do not develop tumors in knee joints when they are 1 × 10^4^ or less in total. Therefore, up to 1 × 10^4^ tumorigenic cells in the implants seem permissible in the case of knee joints.

**Table 1:** Incidence of tumor formation in organs in nude rats over 460 days after knee joint surface defects were implanted with HeLa cells.

Sex	M	M	M	M	F	F	F	F
Number of HeLa cells implanted	1 × 10^5^	1 × 10^4^	1 × 10^3^	1 × 10^2^	1 × 10^5^	1 × 10^4^	1 × 10^3^	1 × 10^2^
**Number of rats**								
Total	4	4	4	4	4	4	4	4
Dead before day 460	0	1**	0	0	0	1	1*	0
Euthanized before day 460 due to knee tumor	3	0	0	0	3	0	0	0
**Number of organs with tumors**								
Liver	0	0	0	0	0	0	0	0
Lung	0	0	0	0	0	0	0	0
Kidney	0	0	0	0	0	0	0	0
Spleen	0	0	0	0	0	0	0	0
Heart	0	0	0	0	0	0	0	0
Brain	0	0	0	0	0	0	0	0
Lymph node	0	0	0	0	0	1	0	0
Pituitary	0	0	0	0	0	0	0	0
Knee	3	0	0	0	3	0	0	0
Subcutaneous	0	0	0	0	0	0	0	0
Testis	0	0	0	0	–	–	–	–

*Brain contusion.

**Cerebral infarction.

### Amount of iPS-Cart safely implanted into articular cartilage defects

In the clinical setting, we are planning to implant many iPS-Cart to fill the defect of articular cartilage. The above cell growth analysis confirmed that 150 mg iPS-Cart contains less than 100 tumorigenic cells, while in vivo examination showed that the malignant cells do not develop tumors when they are less than 1 × 10^4^ in total. The combination of these two results suggests that up to 15 g (= 150 mg × 10^4^ cells/100 cells) of iPS-Cart could be implanted to repair articular cartilage defects in knee joints. Assuming that the articular cartilage thickness in knee joints is around 2.2 mm^26^ and that the specific gravity of iPS-Cart is around 1, 15 g of iPS-Cart would fill 68 cm^2^ of defects, which is larger than most defects seen in knee joint surfaces in clinical settings.

### Limitations of the study

The rationale that we built for calculating the maximum amount of QHJI iPS-Cart that would not produce tumors after implantation has two critical assumptions. One is that tumorigenic cells in iPS-Cart are equivalent to HeLa cells; it is possible that tumorigenic cells in iPS-Cart can be more malignant than HeLa cells. The other is that nude rat knee joints are equivalent to human knee joints; it is possible that a smaller number of HeLa cells form tumors in human knee joints than in nude rat knee joints. In addition, we cannot dismiss the possibility that HeLa cells were rejected by non T-lymphocyte immune cells in nude rats, which would reduce the sensitivity of the test when using nude rat knee joints. In either case, the amount of QHJI iPS-Cart that does not produce tumor after implantation in clinical settings should be decreased. NOG mice have a high degree of immunodeficiency, which would improve the detection sensitivity for transformed cells. However, mice are so small that only a small amount of iPS-Cart can be implanted, lowering the sensitivity. We employed nude rats because they have the largest body size among immunodeficient animals. We put priority on size because larger animals have larger knees that can receive more iPS-Cart, thus, maximizing sensitivity.

Although we employed HeLa cells as a control in this study, other types of malignant cells could theoretically develop from iPSCs due to pluripotency. Chondrosarcoma is one candidate in the case of iPS-Cart, but is rare in joints^27^. Overall, it is difficult to choose one cell line that represents every tumorigenic cell type that can arise from hiPS-Cart. We chose HeLa cells because WHO TRS 878 guidelines and Kuroda et al.^10^ recommend HeLa cells as a control. Further, HeLa cells are one of most widely used and analyzed among tumorigenic cells and as a few as 10 HeLa cells can form tumors in subcutaneous tissue^13^. However, as new information about tumors that tend to develop from iPS-Cart is learned, a different malignant cell line might become a preferred control.

Recent progress in technology can now sequence the whole exons and genome. The knowledge on the genomic stability of iPS-Cart and the presence or absence of mutations in tumor-related genes in iPS-Cart will be gained from this sequencing and will contribute to discussions on the risks of iPS-Cart tumorigenicity in the future.

Finally, regarding the package of preclinical safety tests, tumorigenicity should be examined by the tumorigenicity test in which iPS-Cart is implanted into defects created in the knee joint surface of nude rats. However, in the tumorigenicity test, the amount of iPS-Cart that can be implanted and thus tested is limited, because the knee joints of nude rat are small. Thus, the combination of the tumorigenicity test and the assessment system proposed in this study would provide information on the tumorigenicity of iPS-Cart for judging how much iPS-Cart can be implanted safely into articular cartilage defects in patients in clinical tests.

## Materials and methods

### Cells and culture condition

Human iPS cells (hiPSC): the hiPSC line QHJI was generated from a healthy individual and was a kind gift from K. Okita, M. Nakagawa, and S. Yamanaka (Center for iPS Cell Research and Application (CiRA), Kyoto University, Kyoto, Japan). QHJI was generated from reprogramming human peripheral mononuclear cells by electroporating into them episomal plasmid vectors (pCXLE-hOCT3/4-shp53-F, hSK, hUL, EBNA)^28^. All cells were negative for genome integration.

hiPSC-derived cartilage (iPS-Cart): hiPSCs were chondrogenically differentiated to produce iPS-Cart following a previously described method^14^ with modification. iPS-Cart were generated by chondrogenic differentiation for 12 weeks and maintained in chondrogenic medium (DMEM (Sigma) with 1% ITS, 1% FBS (Thermo), 1 × 10^–4^ M nonessential amino acids (Thermo), 1 mM Na pyruvate (Thermo), 50 units of penicillin, 50 mg/ml of streptomycin, 50 μg/ml ascorbic acid (Nacalai), 10 ng/ml BMP2 (Peprotech), 10 ng/ml TGFβ1 (Peprotech), and 10 ng/ml GDF5 (PTT))^14^ for 0–8 weeks.

HeLa cells: HeLa ATCC CCL-2 cells were obtained from ATCC and maintained in DMEM supplemented with 10% FBS (Invitrogen) and 1% of 100 × penicillin streptomycin (Invitrogen). HeLa cells that had been passaged five times were used for the cell growth analysis and transplantation into nude rats.

### Isolation of cells from iPS-Cart for cell growth analysis

We put various numbers of HeLa cells and 30 mg or 150 mg iPS-Cart into a culture dish and subjected them to digestion in PBS supplemented with 0.25% Trypsin EDTA (Sigma Aldrich) at 37 °C for 1 h, followed by DMEM supplemented by 3 mg/ml collagenase D (Roche) at 37 °C for 3 h. Then the samples were gently pipetted and filtrated (Falcon Cell Strainer 100 µm). The cell suspensions were centrifuged and washed with PBS and plated onto three ϕ100 mm tissue culture dishes (IWAKI) (in the case of 30 mg iPS-Cart) or a 500 cm^2^ cell tray (Sumitomo Bakelite Co., Ltd.) (in the case of 150 mg iPS-Cart) in DMEM with 10% FBS and 1% Penicillin–streptomycin (Life Technologies) and cultured until they reached subconfluency. These cells were subsequently used for the cell growth analysis and senescence examination.

### Cell growth analysis on cells isolated from iPS-Cart

We performed cell growth analysis on the cells isolated from iPS-Cart and contaminated with various numbers of HeLa cells. Cells were passaged as follows when they reached subconfluecy. In the case of 30 mg iPS-Cart, cells in three ϕ100 mm dishes were trypsinized with 0.25% Trypsin EDTA (Sigma Aldrich) for 5 min and mixed together in a tube. The cells were centrifuged (1,000 rpm, 5 min, 22 °C), and the cell pellets were dispersed with Dulbecco’s Modified Eagle Medium (DMEM) containing 10% FBS (Invitrogen) and 1% of 100 × penicillin streptomycin (Invitrogen). Six aliquots were subjected to a cell count by using a Countess II Automated Cell Counter (Thermo), and the average number of cells was calculated. Then 1 × 10^5^ cells were plated onto each of the three ϕ100 mm dishes and cultured. In the case of 150 mg iPS-Cart, cells in a 500 cm^2^ cell tray were trypsinized and subjected to the cell count. Next, 1 × 10^6^ cells were plated onto a 500 cm^2^ cell tray and cultured. The cell number was counted with each passage.

The total cell number and cell growth rate (doubling/day) were calculated as follows:

Total cell number = (N_n+1_/3 × 10^5^ (30 mg iPS-Cart) or 1 × 10^6^ (150 mg iPS-Cart)) × T_n_.

Cell growth rate = [log_2_(N_n+1_/3 × 10^5^ (30 mg iPS-Cart) or 1 × 10^6^ (150 mg iPS-Cart))]/(D_n+1_—D_n_).

T_n;_ Total cell number at passage n, N_n_; Counted live cell number at passage n, D_n_; Days after isolation at passage n.

### Co-culture of HeLa cells with cells isolated from iPS-Cart

5 × 10^3^ HeLa cells were plated in a well of a 6-well plate. Culture inserts (Transwell Inserts, 0.4 μm pore, Corning Inc.) onto which 5 × 10^3^ HeLa cells or 5 × 10^3^ cells isolated from iPS-Cart were seeded were put on the plate. Cells were co-cultured in DMEM supplemented with 10% FBS for 5 days and subjected to microscopic imaging and cell counting. The cell growth rate (doubling/day) was then calculated.

### Examination of cell senescence in iPS-Cart

1 × 10^5^ cells isolated from iPS-Cart were plated onto each of three ϕ100 mm dishes and cultured. When the cells reached subconfluency, the cells were passaged repeatedly as follow: the cells were trypsinized, combined into a tube, and subjected to the cell count. Six aliquots were subjected to the cell count by using a Countess II Automated Cell Counter. Then 1 × 10^5^ cells were re-plated onto each of the three ϕ100 mm dishes and cultured. We performed the experiments in triplicates. We continued the cell culture and the passages until the growth rates of all three samples reached zero.

### Senescence-associated β-galactosidase activity

We examined senescence-associated β-galactosidase activity using the SA-β-gal Staining Cellular Senescence Assay Kit (CELL BIOLABS INC.). After treatment, the cells were stored with PBS containing 20% glycerol.

### mRNA expression analysis

mRNA expression analysis was performed following a previously described method^5^ with modification. iPS-Cart cells were lysed with Qiazol (QIAGEN), and total RNA was extracted with the miRNeasy Kit (QIAGEN). For real-time quantitative RT-PCR, 50 ng of total RNA was reverse transcribed into first-strand cDNA by using ReverTra Ace (Toyobo, Tokyo, Japan) and an oligo(dT)20 primer. The PCR amplification was performed using the KAPA SYBR FAST qPCR Kit Master Mix ABI Prism (KAPA Biosystems, MA, USA). The PCR primers used are listed in Table 2. The RNA expression levels were normalized to the level of GAPDH expression. Results indicate the relative expression of the molecules (control cells = 1).

**Table 2:** Primers used for real-time PCR.

Primer	Sequence
**Human**	
GAPDH forward	AAGCCCATCACCATCTTCCAGGAG
GAPDH reverse	ATGAGCCCTTCCACAATGCCAAAG
P16(INK4A) forward	GTGGACCTGGCTGAGGAG
P16(INK4A) reverse	CTTTCAATCGGGGATGTCTG
P21 forward	TCACTGTCTTGTACCCTTGTGC
P21 reverse	GGCGTTTGGAGTGGTAGAAA

### Transplantation of HeLa cells into defects created in the joint surface of knee joints of nude rats

16 male and 16 female nude rats were used for the experiments. The skin and joint capsules of both knee joints of 4-week-old nude rats (F344/NJcl-rnu rnu/rnu, CLEA Japan, Inc.) were opened. We created an osteochondral defect in the left knee by drilling a hole of 1-mm diameter and 0.5-mm depth at the femoral groove. We transplanted a mixture of HeLa cells and atelocollagen gel (Keeptear, KOKEN CO., LTD.) into the defect. The joint capsule and skin were then closed. The appearance of the rats was observed once a week. The rats were sacrificed when obvious swelling or tumors were observed. Other rats were sacrificed 16 months after the transplantation. After sacrifice, the rats were subjected to an autopsy. The joint capsules were exposed and inspected for the presence of tumors. Then the knees were dissected, fixed in 4% paraformaldehyde, decalcified with EDTA, and embedded in paraffin for the histological analysis.

### Histological and immunohistochemical analysis

Samples were fixed in 4% paraformaldehyde, processed, and embedded in paraffin. Semi-serial sections were prepared and stained with hematoxylin and eosin (HE) or Safranin-O-fast green-iron hematoxylin (Safranin O).

For immunostaining, paraffin-embedded sections were deparaffinized and incubated in 1 mM EDTA buffer (pH 8.0) at 80 °C for 15 min to retrieve the antigen. For the retrieval of HNA antigen, sections were incubated in 1 mM citric acid buffer at 95 °C for 20 min. Then the sections were treated with 10 mg/ml hyaluronidase at room temperature for 40 min. After peroxidase and protein blocking with 3% hydrogen peroxide solution and serum-free protein in buffer, the sections were incubated with primary antibody overnight at 4 °C.

The primary antibodies used were as follows: mouse anti-HNA (Invitrogen, 1: 500) and rabbit anti-human vimentin (Abcam, 1:200). The primary antibodies were detected with a CSA II Biotin-free Tyamide Signal Amplification System Kit (Agilent Technologies, CA, USA) and DAB as a chromogen.

For the immunofluorescent staining of HeLa cells, 1 × 10^6^ cells were plated on a Nunc Lab-Tek Chamber Slide (Merck), fixed with 4% paraformaldehyde for 15 min, treated with 0.5% Triton X-100 (Merck) for 15 min, and then treated with the primary antibody of HPV18E7 (Abcam, 1:250). Immune complexes were detected using secondary antibodies conjugated to Alexa Fluor 488 (Life Technologies, 1:1,000) in Can Get signal A (Toyobo, Tokyo, Japan). Fluoroshield Mounting Medium with DAPI (Abcam) was used.

### Statistical analysis

The data are shown as the means and standard deviations. In this study, we used one-way ANOVA (analysis of variance) with Tukey's HSD tests for multiple comparisons. P-values < 0.05 were considered statistically significant.

### Ethics statement

All methods were carried out in accordance with relevant guidelines and regulations. Experiments using recombinant DNA was approved by the Recombinant DNA Experiments Safety Committee of Kyoto University. Research involving human subjects was approved by the Ethics Committee of Kyoto University. Written informed consent was obtained from each donor. All animal experiments were approved by the institutional animal committee of Kyoto University.

## References

[1] Huey DJ, Hu JC, Athanasiou KA (2012). Unlike bone, cartilage regeneration remains elusive. Science (New York).

[2] Castro-Vinuelas R (2018). Induced pluripotent stem cells for cartilage repair: Current status and future perspectives. Eur. Cell Mater..

[3] Chesterman PJ, Smith AU (1968). Homotransplantation of articular cartilage and isolated chondrocytes. An experimental study in rabbits. J. Bone Joint Surg. Br..

[4] Adkisson HD (2010). Immune evasion by neocartilage-derived chondrocytes: Implications for biologic repair of joint articular cartilage. Stem Cell Res..

[5] Kimura T, Yamashita A, Ozono K, Tsumaki N (2016). Limited immunogenicity of human induced pluripotent stem cell-derived cartilages. Tissue Eng. Part A.

[6] Erdmann J (2011). ISTO Technologies aims to rescue damaged joints. Chem. Biol..

[7] Farr J, Tabet SK, Margerrison E, Cole BJ (2014). Clinical, radiographic, and histological outcomes after cartilage repair with particulated juvenile articular cartilage: A 2-year prospective study. Am. J. Sports Med..

[8] Tompkins M (2013). DeNovo NT allograft. Oper. Tech. Sports Med..

[9] Adkisson HD (2010). The potential of human allogeneic juvenile chondrocytes for restoration of articular cartilage. Am. J. Sports Med..

[10] Kuroda T, Yasuda S, Sato Y (2013). Tumorigenicity studies for human pluripotent stem cell-derived products. Biol. Pharm. Bull..

[11] Ito E (2019). Tumorigenicity assay essential for facilitating safety studies of hiPSC-derived cardiomyocytes for clinical application. Sci. Rep..

[12] Kuroda T (2012). Highly sensitive in vitro methods for detection of residual undifferentiated cells in retinal pigment epithelial cells derived from human iPS cells. PLoS ONE.

[13] Kusakawa S (2015). Characterization of in vivo tumorigenicity tests using severe immunodeficient NOD/Shi-scid IL2Rgamma(null) mice for detection of tumorigenic cellular impurities in human cell-processed therapeutic products. Regener. Therapy.

[14] Yamashita A (2015). Generation of Scaffoldless hyaline cartilaginous tissue from human iPSCs. Stem Cell Rep..

[15] Yamashita A (2014). Statin treatment rescues FGFR3 skeletal dysplasia phenotypes. Nature.

[16] Hayflick L (1965). The limited in vitro lifetime of human diploid cell strains. Exp. Cell Res..

[17] Hayflick L (1985). The cell biology of aging. Clin. Geriatr. Med..

[18] Hayflick L, Moorhead PS (1961). The serial cultivation of human diploid cell strains. Exp. Cell Res..

[19] Hasebe-Takada N (2016). Application of cell growth analysis to the quality assessment of human cell-processed therapeutic products as a testing method for immortalized cellular impurities. Regener. Therapy.

[20] Kono K (2015). Characterization of the cell growth analysis for detection of immortal cellular impurities in human mesenchymal stem cells. Biologicals.

[21] *European medicines Agency EMEA/724428/2009 assessment report for ChondroCelect.*, (2009).

[22] Organization WH (2013). Recommendations for the evaluation of animal cell cultures as substrates for the manufacture of biological medicinal products and for the characterization of cell banks. World Health Organ. Tech. Rep. Ser..

[23] Dixon D, Heider K, Elwell MR (1995). Incidence of nonneoplastic lesions in historical control male and female Fischer-344 rats from 90-day toxicity studies. Toxicol. Pathol..

[24] Dinse GE, Peddada SD, Harris SF, Elmore SA (2010). Comparison of NTP historical control tumor incidence rates in female Harlan sprague dawley and fischer 344/N rats. Toxicol. Pathol..

[25] Haseman JK, Hailey JR, Morris RW (1998). Spontaneous neoplasm incidences in Fischer 344 rats and B6C3F1 mice in two-year carcinogenicity studies: A National Toxicology Program update. Toxicol. Pathol..

[26] Aigner T (2010). Histopathology atlas of animal model systems—overview of guiding principles. Osteoarthritis Cartilage.

[27] Unni KK, Inwards CY (2020). Dahlin’s Bone Tumors.

[28] 28Okita, K. *et al.* A more efficient method to generate integration-free human iPS cells. *Nat Meth* **8**, 409–412, https://www.nature.com/nmeth/journal/v8/n5/abs/nmeth.1591.html#supplementary-information (2011). 10.1038/nmeth.1591 21460823

